# Outcomes of intravesical Bacillus Calmette-Guerin in patients with non-muscle invasive bladder cancer: a retrospective study in Australia

**DOI:** 10.3389/fruro.2024.1309532

**Published:** 2024-02-14

**Authors:** Chamodi Pillippu Hewa, Stephen Della-Fiorentina, Kayvan Haghighi, Wei Chua, Peey-Sei Kok

**Affiliations:** ^1^ School of Medicine, Western Sydney University, Sydney, NSW, Australia; ^2^ Medical Oncology, Southern Highlands Cancer Centre, Bowral, NSW, Australia; ^3^ Macarthur Cancer Therapy Clinic, Campbelltown Hospital, Sydney, NSW, Australia; ^4^ Macarthur Urology, Campbelltown Private Hospital, Sydney, NSW, Australia; ^5^ Department of Urology, Campbelltown Hospital, Sydney, NSW, Australia; ^6^ Liverpool Cancer Therapy Centre, Liverpool Hospital, Sydney, NSW, Australia; ^7^ School of Medicine, University of New South Wales, Sydney, NSW, Australia; ^8^ National Health and Medical Research Council (NHMRC) Clinical Trials Centre, University of Sydney, Sydney, NSW, Australia

**Keywords:** intravesical BCG, NMIBC, disease-free survival, bladder cancer, TURBT, overall survival

## Abstract

**Introduction:**

Induction intravesical Bacillus Calmette-Guerin (BCG) followed by maintenance after transurethral resection of bladder tumor, is the standard adjuvant therapy for high-risk non-muscle invasive bladder cancer (NMIBC). There is sparse evidence on the practice of intravesical BCG in Australia. Our aim was to determine the outcomes of intravesical BCG therapy in NMIBC in Southwestern Sydney.

**Methods:**

This was a multi-center retrospective audit of NMIBC patients who received intravesical BCG between January 2008 and June 2020. Data was collected across six tertiary hospitals in South Western Sydney. Primary outcome was disease-free survival (DFS). Secondary outcomes were overall survival (OS), BCG induction and maintenance rates.

**Results:**

Of the 200 eligible patients over 12.5 years, median age was 77 years and 83% were male. Of these, 55%, 4.5%, 35% and 5% were Tis, Ta, T1 and unknown stage, respectively. All patients received induction BCG and 56% received maintenance BCG (range 3-36 months). Completion rate of induction BCG was 91%. Only 9% ceased treatment due to intolerance. The median duration of cystoscopy follow-up was 17 months. After a median follow-up time of 37 months, 55% developed recurrence (29% non-muscle invasive, 32% muscle-invasive disease, 8% distant metastasis). The 1-year and 5-year DFS rates were 72% and 41% (median DFS: 39 months). The 1-year and 5-year OS rates were 98% and 87% (median OS: not reached).

**Conclusion:**

The DFS and OS rates were comparable to previous literature. This provides real-world data to assist future clinical trials in NMIBC.

## Introduction

Bladder cancer is the tenth most common cancer in Australia, affecting more men than women (1). In 2017 alone, 2743 Australians were diagnosed with bladder cancer where 832 new cases were from New South Wales (NSW) (2, 3).

Approximately 75% of bladder cancers are categorized as non-muscle invasive bladder cancers (NMIBC) (4). NMIBC is a definition used for three subtypes of tumors which are appropriate for transurethral resection: tumors confined to the mucosa or invading the lamina propria (Ta, T1) and flat, high-grade tumors confined to the mucosa (Tis or carcinoma *in situ*/CIS) (4). The risk of disease-free survival (DFS) for NMIBC depends on the histopathological stage and risk group. The prognosis of Ta and T1 tumors is based on the European Organization of Research and Treatment for Cancer (EORTC) scoring model which considers the number of tumors, tumor size, prior recurrence rate, T category, presence of concurrent CIS and the tumor grade (4). Without any treatment, patients with Ta disease have a lower risk of progression (6 to 28%) in comparison to patients with Tis disease who have a 45% risk of progression to invasive bladder cancer (4, 5).

Numerous clinical trials and multiple international guidelines (European Association of Urology) have recommended transurethral resection of the bladder tumor (TURBT) as the standard curative treatment for NMIBC (4, 6). The recurrence rate of Ta and T1 tumors remains high despite TURBT, of up to 56% in 1 year (4, 6). Several meta-analyses have shown that intravesical BCG therapy significantly reduces the risk of tumor recurrence in patients with NMIBC, compared to TURBT alone and intravesical chemotherapy in combination with TURBT (6–8). Therefore, adjuvant intravesical BCG therapy is indicated in those with high risk of recurrence (T1 G3, Tis or multiple recurrent Ta G1-2 tumors greater than 3cm) (4, 9). The standard course of induction intravesical BCG is administered weekly over a six week period followed by maintenance treatment (4). While most meta-analyses suggest that maintenance BCG may reduce DFS and OS, there is conflicting data as to whether a longer period of maintenance treatment provides a statistically significant improvement in OS (10–13). Installation of maintenance BCG also ranged from quarterly, monthly, 3-weekly and 6-weekly schedules, but a critical review of maintenance BCG showed that the 3-week schedule used in the SWOG trial had reduced recurrence and progression (14). Many guidelines recommend maintenance BCG with 3 weekly instillations for up to three years in patients with high risk NMIBC, but this practice varies widely between countries and centers (4, 9). The European Association of Urology guidelines recommend using full dose intravesical BCG in patients with high risk NMIBC for a minimum of one year and the additional beneficial effect of the second and third years of maintenance treatment should be weighed against its costs, side effects and access to BCG (4).

In Australia, the five-year survival for bladder cancer has dropped from 68% to 58% in the past 30 years, despite a decreasing incidence (3, 15). Southwestern Sydney is one of the largest health districts in NSW, with an estimated population of approximately 966,540 residents, which continues to grow (16, 17). This region has the highest incidence of bladder cancer in New South Wales (29.6%) and a higher rate of unemployment (7.6%) compared with the state average of only 6.3% (17, 18). The median total income per annum in Southwestern Sydney in 2018 was $48,254 compared to the state median income of $49,805 (16). Therefore, it is our research interest to better understand the trend of bladder cancer, particularly with the high incidence of smoking and socioeconomical disadvantage in this region, and its potential impact on the receipt of standard of care treatment.

In this study, we aimed to determine the outcomes and practice of intravesical BCG therapy in patients with NMIBC within Southwestern Sydney.

## Materials and methods

This was a retrospective multi-center cohort study across six hospitals in Southwestern Sydney on patients diagnosed with NMIBC, and treated with intravesical BCG, between January 2008 and February 2020. Inclusion criteria compromised of patients of all ages and genders, who were diagnosed with NMIBC (high grade Ta, T1, CIS stage) and received TURBT followed by induction BCG. These patients were identified from the electronic medical record (EMR) databases and diagnosis of NMIBC was based on a combination of cytology, and histopathological results, while risk stratification of patients was based on histopathology alone. All the patients included in the study had a diagnosis of NMIBC. The exclusion criteria were as follows: previous history of muscle invasive cancer, or muscle- invasive cancer at diagnosis, patients who received previous systemic chemotherapy or radiotherapy for bladder cancer, patients with metastatic disease at diagnosis, upper urinary tract urothelial tumors and non-urothelial bladder cancer and patients without initial cystoscopies. Completion of induction BCG was defined by the completion of receiving the full 6-weekly cycles of induction BCG.

The delivery of intravesical BCG therapy was centralized at one of the six hospitals (Southern Highlands Cancer Centre), under the medical oncology team. Cystoscopies were conducted by the urologists across all six public hospitals and two private hospitals in Southwestern Sydney. The post BCG surveillance protocol included cystoscopy and biopsy for all patients who were not receiving maintenance treatment and flexible cystoscopies for patients receiving maintenance treatment. Maintenance treatment was delivered according to the standard guidelines which recommend 3-weekly intravesical BCG instillations at 3, 6, 12, 18, 24, 30 and 36 months for patients with high-risk disease (4).

Baseline characteristics data collected included: age, sex, stage and grade of tumor and date of diagnosis of NMIBC. Treatment data included the number of patients who received TURBT, received induction BCG, maintenance BCG and rechallenged induction BCG at recurrence.

Additional data recorded included dates of follow up cystoscopies and adverse effects of BCG.

The primary endpoint was disease-free survival (DFS), which was measured from the date of diagnosis to the date of first recurrence or death. Secondary endpoints included overall survival (OS), rate of completion of induction BCG, adverse events (AEs) of BCG and frequency of cystoscopy follow-up. All urinary tract infections were based on documented positive urine cultures. OS was calculated from the date of diagnosis to the date of death. All patients were followed up to 18^th^ of March 2021.

### Statistical analysis

All statistical analyses were conducted using STATA 15 software. Descriptive statistics were applied to analyze patient baseline characteristics. OS and DFS were estimated by the Kaplan Meier method. Uni- and multi-variate cox proportional hazard models were employed to evaluate the effect of prognostic variables (age, sex, maintenance BCG, and stage) in DFS and OS. These results were described using hazard ratio (HR) and 95% confidence intervals (CI).

This quality assurance study was reviewed by the quality assurance team at Campbelltown Hospital and was deemed that it would not pose additional ethical risks to patients and met the guidance provided in Section 2 (e) of the National Health and Medical Research Council “Ethical Considerations in Quality Assurance and Evaluation Activities”. It was recommended that it did not require further review from the Human Research Ethics Committee.

## Results

**Figure 1:**
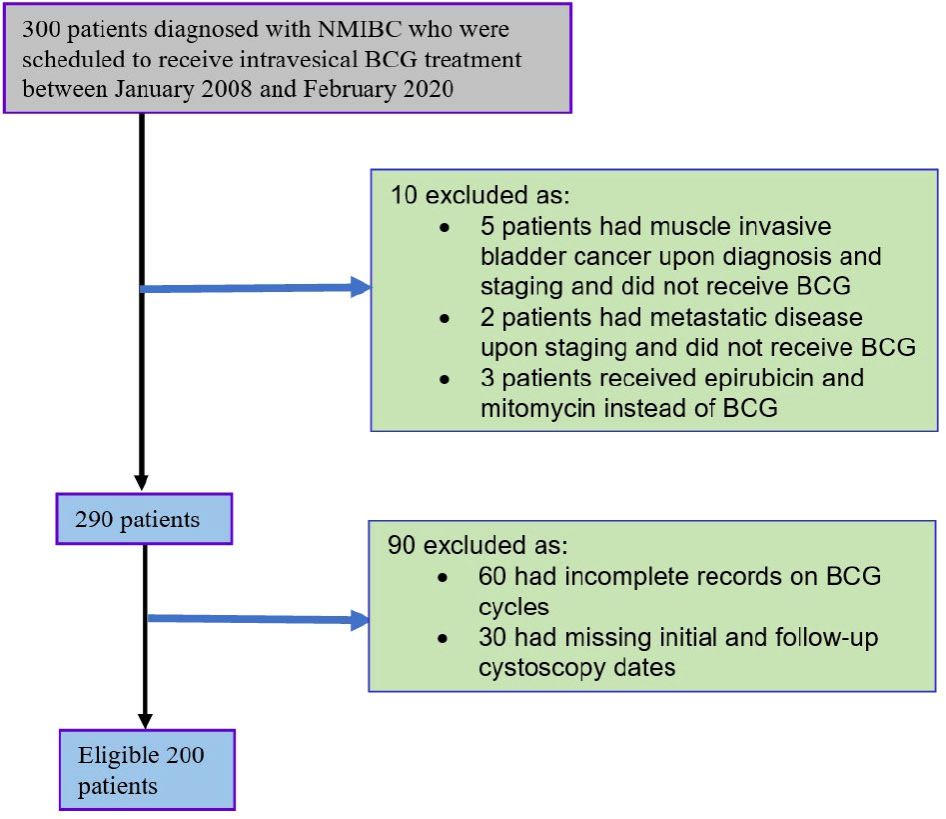
Study design with inclusion and exclusion criteria.

Between the 1^st^ of January 2008 and the 11^th^ of February 2020, 300 patients were diagnosed with NMIBC in six hospitals in South Western Sydney. One hundred patients were excluded (60 had incomplete BCG records, 30 had missing initial cystoscopy reports, 5 had muscle invasive bladder cancer upon staging, 3 patients were given intravesical epirubicin and intravesical mitomycin C (MMC) instead of intravesical BCG and two patients had metastatic disease upon staging, (see **Figure 1**). All patients received the standard dose of BCG (500 million CFU).

In total, 200 patients with NMIBC who received BCG treatment were included in this analysis. Of the 200 patients included, the diagnoses made by grading are listed in **Table 1**. Fifty-three of these patients developed concurrent non muscle invasive disease from intermediate risk low grade disease and received BCG at that time and are captured in this cohort. We did not have information on the percentage of patients with recurrent low-grade disease who did not receive BCG.

**Table 1:** Baseline characteristics results.

Baseline characteristics	All patients (n=200)	%
Age, years		
**Median**	77	
**Range**	46-98	
Sex		
**Male**	165	(83)
**Female**	35	(17)
Clinical stages		
**Tis**	109	(55)
**Ta**	9	(4.5)
**T1**	70	(35)
**Unknown**	10	(5)
Risk		
**Grade 1**	9	(4.5)
**Grade 2**	14	(7)
**Grade 3**	147	(73.5)
**Unknown**	30	(15)
**Recurrence of NMIBC with intermediate risk low grade disease**	53	(27)

NMIBC, Non-muscle invasive bladder cancer.

### Baseline characteristics

The median age was 77 years (range, 46-98 years) and the study population consisted of 83% (165/200) males and 17% (35/200) females. Of the 200 patients, 89% (177/200) had clear documentation on clinical staging at diagnosis. A majority of these patients were TIS (55%, n=109) and grade 3 (74%, n=147), while the clinical stages for 5% of patients (10/200) were unknown. Of the patients were TIS, 53 patients had concomitant Tis with Ta and 40 patients had concomitant Ta with TIS. The baseline characteristics are summarized in **Table 1**.

### BCG treatment

All patients were planned to receive a 6-week course of induction BCG. Of these, 91% (182/200) completed weekly cycles of induction BCG for 6 weeks; only 9% (18/200) failed to complete this. (**Table 2**). Of the two hundred patients, 56% (112/200) received maintenance BCG after completing induction therapy. Of the 112 patients, 38 patients received at least 6 months of maintenance treatment and 14 patients received at least 12 months of maintenance treatment. Nineteen patients who received 6 months of maintenance treatment developed recurrence subsequently, while only five patients who received 12 months of maintenance treatment developed a further recurrence. The duration of initial maintenance treatment after induction BCG, ranged from 3 to 36 months.

**Table 2:** Recurrence rate.

	All patients (n=200)	%
**Recurrence rate**	111/200	(55)
Non-muscle invasive	32	(29)
Invasive	79	(71)
Muscle invasive	63	(80)
Distant invasive	16	(20)

Muscle invasive disease is defined as recurrence within and beyond T2 staging where the tumor invades the muscle layer of the bladder. Distant invasive disease is defined as recurrence extending to distant organs.

Thirty-two patients had recurrence of non-muscle invasive disease and received further TURBT. Of these, 83% (27/32) received re-challenged induction BCG and only 13 patients received maintenance subsequently.

### Cystoscopy follow up

Patients of this study attended the urology service provided by 12 urologists. 72% (143/200) were followed up by their treating urologists post BCG in Southwestern Sydney and follow up information was not available in 28% of patients. Based on the available information, all patients received repeat resection prior to the commencement of induction BCG and 48% of the 200 patients received re-resection afterwards. Median duration of cystoscopy follow up was 17 months (range of 1-246 months).

### Survival analyses

**Figure 2:**
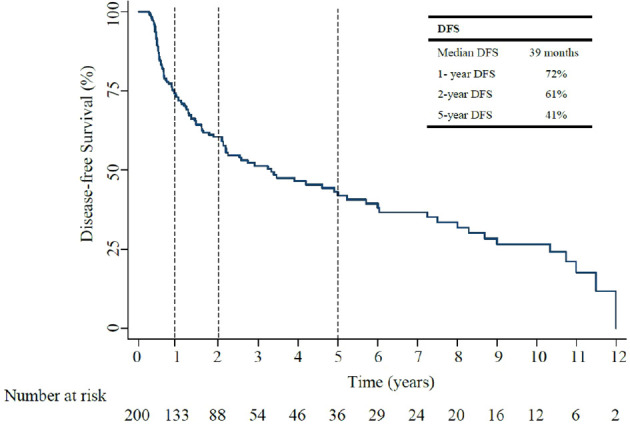
Kaplan-Meier Disease Free Survival (DFS) after BCG treatment.

Over a median follow up of 37 months, (IQR 20.2-71.6), median DFS was 39 months (95% CI, 15.6-24.5: **Figure 2**). The 1-year, and 5-year DFS rates were 72%, and 41%. In patients who had recurrent non muscle invasive disease, median DFS was 40 months, with 1-year and 5-year DFS rates of 72% and 43% (**Appendix Table 1**). In patients who had muscle invasive disease and metastatic disease, median DFS was 10 months, with 1-year and 5-year DFS rates of 44% and 6% (**Appendix Table 1**). Of the 200 patients, 55% (111/200) developed disease recurrence [29% non-muscle invasive and 71% invasive disease (including muscle invasive disease and distant metastasis)]. Of those who had invasive disease recurrence, 80% had muscle invasive disease recurrence (defined as recurrence within and beyond the muscle layer of the bladder, and/or within adjacent lymph nodes) and 20% distant metastasis.

**Figure 3:**
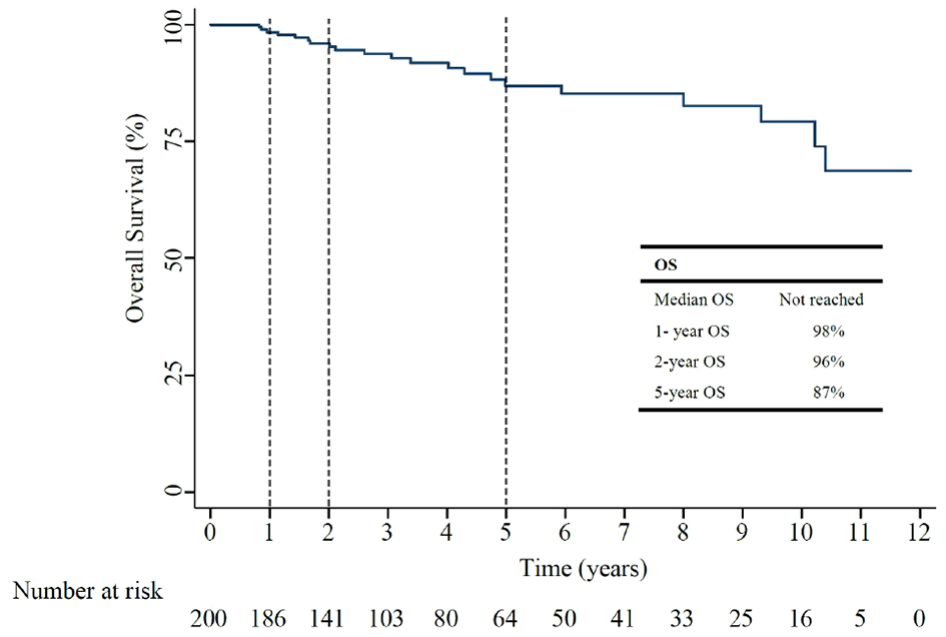
Overall Survival (OS) curve after BCG treatment.

The median OS for all included patients was not reached (**Figure 3**). The 1-year, and 5-year OS rates were 98%, and 87%, as shown in **Table 2**. In patients who had recurrent non-muscle invasive disease, median OS was not reached, with 1-year and 5-year OS rates of 100% and 91% (**Appendix Table 2**). In patients who had muscle invasive disease and distant metastasis, the 1-year and 5-year OS rates of 98% and 84% (**Appendix Table 2**).

In the subgroup analysis, the median DFS for T1 was 10 months while the median DFS for CIS/Ta was 13 months. The recurrence rates were 50% vs 58% respectively and the median OS in both groups were not reached.

### Prognostic variables

In univariate analyses, none of the baseline characteristics (age, gender, stage of tumor) or having maintenance were prognostic of DFS and OS (**Table 3**). In multivariate analyses, none of the baseline characteristics (age, gender, stage of tumor) or having maintenance BCG were prognostic of DFS and OS.

**Table 3:** Univariate and Multivariate analyses.

Prognostic variables	Hazard ratio	Univariate analysis	Hazard Ratio	Multivariate analysis
Disease Free Survival				
**Age**	0.98 (0.96-1.00)	P = 0.07	0.99 (0.97-1.01)	P=0.17
**Gender (male vs female)**	0.96 (0.58-1.58)	P = 0.87	0.97 (0.58-1.64)	P=1.64
**Stage** **(Ta, Tis, T1)**	0.99 (0.66-1.50)	P = 0.96	1.02 (0.68-1.54)	P=0.92
**Maintenance BCG (yes vs no)**	1.19 (0.81-1.75)	P = 0.37	1.09 (0.73-1.64)	P=0.67
Overall Survival				
**Age**	1.04 (0.99-1.08)	P = 0.12	1.04 (0.98-1.10)	P=0.16
**Gender (male vs female)**	2.36 (0.55-10.22)	P = 0.25	3.86 (0.51-29.2)	P=0.19
**Stage** **(Ta, Tis, T1)**	1.86 (0.49-7.06)	P = 0.36	1.12 (0.46-6.84)	P=0.41
**Maintenance BCG (yes vs no)**	0.79 (0.33-1.90)	P = 0.59	0.97 (0.37-2.53)	P=0.96

BCG, Bacillus Calmette Guerin.

### Adverse events

Intravesical BCG was generally well tolerated. Treatment-related AEs were experienced by 18% (**Table 4**), most common being urinary tract infection followed by hematuria. Of these, only 18 patients (9%) ceased BCG treatment, of which 33% (6/18) stopped due to severe sepsis, 28% (5/18) stopped due to recurrent infection, 22% (4/18) stopped due to social reasons and 17% (3/18) stopped due to other medical reasons. None of the patients reported systemic BCG sepsis as an adverse event of BCG.

**Table 4:** Adverse events of BCG.

Treatment-related AEs	36	(18)
Infection	18/36	(50)
Hematuria/bleeding	13/36	(36)
Pain	5/36	(14)
**Cessation of BCG**	18/200	(9)

AE, adverse effects; BCG, Bacillus-Calmette Guerin.

## Discussion

Our study summarized the outcomes of patients with NMIBC over 12 years in Southwest Sydney. All patients received intravesical BCG and 56% of patients received maintenance therapy. Approximately half of these patients developed a subsequent recurrence, of which 29% were non-muscle invasive disease and 71% were invasive disease (80% muscle invasive disease, localized within and beyond the muscle layer of the bladder and adjacent lymph nodes and 20% distant metastasis). Most of the patients with recurrent non-muscle invasive disease received reintroduction of induction BCG and 41% received maintenance subsequently. The 5-year disease-free survival and 5-year overall survival in our study were 41% and 87%. Very few patients stopped due to BCG-related AEs, most commonly related to cystitis.

Intravesical BCG has been the standard of care for NMIBC patients in the last 20 years. The DFS and OS outcomes of the patients from our cohort were comparable to that from previous literature (14, 19). A systematic review of multiple RCTs of intravesical BCG reported 2-year recurrence-free survival rates ranged between 54% and 89% (19). A 2009 meta-analysis of individual patient data comparing intravesical BCG vs MMC reported a recurrence-free rate of 43% and an OS of 79.7% in the BCG group, over a median follow-up of 4.4 years (20).

In our study, only 56% of patients received maintenance after initial diagnosis and 41% received maintenance at recurrence of non-muscle invasive disease. The recurrence rate at 55% was higher than expected likely contributed by high incidence of smoking, poor socioeconomic status, and health literacy in this demographic population from the Southwest area of Sydney, Australia. Another major factor is likely from the lack of maintenance BCG treatment. Whilst we were unable to demonstrate this research piece due to its limitation as a retrospective review without control group, our urologists have raised concerns that many patients chose to have surveillance cystoscopy but opted not to have maintenance treatment due to poor compliance.

Multiple meta-analyses of RCTs have indicated that at least 1 year of maintenance treatment prolonged OS and DFS (21–23). However, in the EORTC-GU RCT, no OS benefit was found in patients who received 3 years vs 1 year of the full dose of maintenance BCG (74% vs 71.3%), despite reduced recurrences in high-risk patients (HR:0.75; 95% CI 0.59-0.94; p=0.01) (10). The SWOG RCT in patients with recurrent superficial disease reported doubled recurrence-free survival (median of 76.8 months vs 35.7 months) in patients receiving maintenance 3 weeks (given 3, 6, 12, 18-, 24-, 30- and 36-months post induction) vs no maintenance BCG, with no significant OS difference (14). This has formed the standard schedule of current maintenance BCG in many countries. One should note the selection bias in these patients as maintenance BCG was given to those who have not progressed after induction BCG. Overall, maintenance BCG improved DFS, particularly in high-risk patients. Compiling these conflicting results, guidelines from AUA, EAU and Canadian Urology Association support at least 1 year of maintenance intravesical BCG (8, 10, 21).

### Implications of research

Our findings provide valuable clinical implications for future research. To our knowledge, this study is one of the few large, multicenter retrospective studies looking at the outcomes of intravesical BCG therapy in high risk non muscle invasive bladder cancer, within Australia. We found that the outcomes of NMIBC in patients in Southwestern Sydney are similar to that from previous literature, despite the higher incidence of smokers and lower socioeconomic status. Our data provides real-world data, which is useful for oncologists to understand the use of BCG and maintenance therapy within Southwestern Sydney. The variation in the range of maintenance treatment from 3-36 months, reduced number of patients receiving maintenance, and a median cystoscopy follow up period of only 17 months in our study, may have contributed to a high recurrence rate of 55%. Therefore, longer cystoscopy follow-up and longer maintenance therapy may further improve the prognosis of high-risk NMIBC patients.

After almost 20 years of paucity of evidence or progress in this disease setting, there has been promising data for novel agents in NMIBC, particularly in BCG-refractory setting (24). In the single-arm phase 2 trial of pembrolizumab (KEYNOTE-057), a programmed cell death protein 1 (PD-1) inhibitor given in patients with high-risk BCG-unresponsive CIS showed that 41% had a complete response at three months, and 46% remained in complete response for 12 months (24). Despite the FDA approval, it is important to note that without an RCT comparing pembrolizumab and standard of care, the true clinical benefit in survival is unclear. Furthermore, the financial toxicity of such treatment should not be underestimated (25).

With further development in personalized medicine, one should explore the molecular alterations of bladder cancer. The Cancer Genome Atlas Research Network reported on statistically significant recurrent mutations in 32 genes, based on the analysis of 131 urothelial carcinomas (26). This analysis found therapeutic targets in 69% of urothelial tumors, raising the question as to whether targeted therapies can be used for NMIBC in the future (26).

### Strengths and limitations

The major strengths of our study are that it provides real world data on the practice of BCG and outcomes of NMIBC in Australia, across multiple centers. Our large sample size, consisting of 200 patients provided long-term data, reflecting on the practice of NMIBC management in the Southwest Sydney.

However, the retrospective nature of our study had several limitations. Our data relied on the documentation provided by individual clinicians on electronic medical records. Missing information on the exact BCG cycle dates prior to 2018, missing information on follow up cystoscopies, and smoking history may affect the results of our study. We excluded 90 patients due to missing BCG treatment records and missing initial cystoscopy information. These were patients who received treatment in the private sector prior to 2018 and had missing records on the electronic medical records database. The variants of histology (such as squamous cell, small cell and micropapillary components) were not recorded and may affect the outcome of these patients. We also could not report on the tumor response post BCG as this relied on consistent reporting from cystoscopy follow up. The reduced percentage of patients receiving maintenance treatment and lack of follow up cystoscopy information on patients who visited private urologists, are some potential factors contributing to the lack of significant prognostic variables predicting DFS and OS. Our study was subjected to selection bias as we lacked a control group comprising of patients who did not receive intravesical induction BCG as a comparison to patients who received induction BCG. We acknowledge that 28% of patients lacked information on follow up cystoscopies and the results of our study need to be interpreted taking this into consideration. Although this may affect the outcomes of disease-free survival, it is unlikely to affect overall survival.

## Conclusions

The use of intravesical BCG therapy is the standard treatment of choice in fully resected high risk non-muscle invasive bladder cancer (4). Although our demonstrated DFS and OS rates are comparable with previous literature, the high overall recurrence rate of 55% in this population warrants more research into new effective therapies.

## Data availability statement

The raw data supporting the conclusions of this article will be made available by the authors, without undue reservation.

## Ethics statement

The studies involving humans were approved by Quality Assurance Team at Campbelltown Hospital. The studies were conducted in accordance with the local legislation and institutional requirements. Written informed consent for participation was not required from the participants or the participants’ legal guardians/next of kin in accordance with the national legislation and institutional requirements.

## Author contributions

CP: Data curation, Formal Analysis, Investigation, Resources, Writing – original draft, Writing – review & editing. SD-F: Data curation, Supervision, Writing – review & editing, Conceptualization. WC: Writing – review & editing. KH: Writing – review & editing. P-SK: Writing – review & editing, Conceptualization, Formal Analysis, Supervision, Validation, Writing – original draft, Resources, Methodology.
