# Supplementary material for: Outcomes of intravesical Bacillus Calmette-Guerin in patients with non-muscle invasive bladder cancer: a retrospective study in Australia
Source: Front Urol. 2024 Feb 14;4:1309532. doi: 10.3389/fruro.2024.1309532 (PMC12327299; doi:10.3389/fruro.2024.1309532)
Supplement: Supplementary file 1 [file Table_1.docx]

# Appendices

**Appendix 1.** Disease free survival in non-muscle invasive and invasive disease …………………..…21

**Appendix 2.** Overall survival in non-muscle invasive and invasive DFS………………………………22

# Appendix Table 1

## Disease-Free Survival in non-muscle invasive, muscle invasive disease and metastases

| **Recurrence rate** | **%** |
| --- | --- |
| **Non-muscle invasive disease** | 29 (32/111) |
| Median DFS, months | 40 |
| 1-year DFS | 72 |
| 5-year DFS | 43 |
| **Muscle invasive disease and metastases** | 71 (79/111) |
| Median DFS, months | 10 |
| 1-year DFS | 44 |
| 5-year DFS | 6 |

**Abbreviations: DFS, disease free survival**

**Appendix Table 2**

## Overall survival in non-muscle invasive, muscle invasive disease and metastases

| **Overall Survival** |  |
| --- | --- |
| **Non-muscle invasive OS (%)** |  |
| Events | 29 (32/111) |
| 1-year | 100 |
| 5-year | 91 |
| **Muscle invasive and metastases OS (%)** |  |
| Events | 71 (79/111) |
| 1-year | 98 |
| 5-year | 84 |

**Abbreviations: OS, Overall survival**
